# Interplay between Cultured Human Osteoblastic and Skeletal Muscle Cells: Effects of Conditioned Media on Glucose and Fatty Acid Metabolism

**DOI:** 10.3390/biomedicines11112908

**Published:** 2023-10-27

**Authors:** Ngoc Nguyen Lunde, Nimo Mukhtar Mohamud Osoble, Andrea Dalmao Fernandez, Alfreda S. Antobreh, Abbas Jafari, Sachin Singh, Tuula A. Nyman, Arild C. Rustan, Rigmor Solberg, G. Hege Thoresen

**Affiliations:** 1 Section for Pharmacology and Pharmaceutical Biosciences, Department of Pharmacy, University of Oslo, 0316 Oslo, Norway; n.n.lunde@farmasi.uio.no (N.N.L.); n.m.m.osoble@farmasi.uio.no (N.M.M.O.); a.d.fernandez@farmasi.uio.no (A.D.F.); a.c.rustan@farmasi.uio.no (A.C.R.); rigmor.solberg@farmasi.uio.no (R.S.); 2 Department of Cellular and Molecular Medicine, University of Copenhagen, 2200 Copenhagen, Denmark; ajafari@sund.ku.dk; 3 Department of Immunology, Oslo University Hospital, Rikshospitalet, University of Oslo, 0372 Oslo, Norway; sachin.singh@medisin.uio.no (S.S.); t.a.nyman@medisin.uio.no (T.A.N.); 4 Department of Pharmacology, Institute of Clinical Medicine, University of Oslo, 0316 Oslo, Norway

**Keywords:** energy metabolism, myokines, osteoblastic cells, osteokines, primary human myotubes

## Abstract

The interplay between skeletal muscle and bone is primarily mechanical; however, biochemical crosstalk by secreted mediators has recently gained increased attention. The aim of this study was to investigate metabolic effects of conditioned medium from osteoblasts (OB-CM) on myotubes and vice versa. Human skeletal muscle cells incubated with OB-CM showed increased glucose uptake and oxidation, and mRNA expression of the glucose transporter (*GLUT*) *1*, while fatty acid uptake and oxidation, and mRNA expression of the fatty acid transporter *CD36* were decreased. This was supported by proteomic analysis, where expression of proteins involved in glucose uptake, glycolytic pathways, and the TCA cycle were enhanced, and expression of several proteins involved in fatty acid metabolism were reduced. Similar effects on energy metabolism were observed in human bone marrow stromal cells differentiated to osteoblastic cells incubated with conditioned medium from myotubes (SKM-CM), with increased glucose uptake and reduced oleic acid uptake. Proteomic analyses of the two conditioned media revealed many common proteins. Thus, our data may indicate a shift in fuel preference from fatty acid to glucose metabolism in both cell types, induced by conditioned media from the opposite cell type, possibly indicating a more general pattern in communication between these tissues.

## 1. Introduction

The interplay between bone and skeletal muscle is of high interest, as the organs are closely connected through the musculoskeletal system. The mechanical interaction between muscle and bone is well described (reviewed by [1]), and dysregulation of one organ affects the other organ, for example, muscle atrophy results in osteoporosis [2]. Mechanical loading contributes to preserving the mechanical integrity of bone. It has been suggested that skeletal muscle contraction is necessary for the recovery of bone loss (reviewed by [2]). In addition to the mechanical interaction, recent studies have shown that skeletal muscle and bone interact via biochemical crosstalk, where skeletal muscles are able to secrete soluble molecules regulating bone and vice versa [2,3]. Skeletal muscles produce and secrete myokines essential in skeletal muscle crosstalk with other tissues, including cytokines and other proteins [4,5,6], and shown to have anabolic and catabolic effects on bones [7]. Osteokines secreted from bone-forming osteoblastic cells are able to exert induction of muscle anabolism or catabolism, depending on the secreted molecule [1,2]. As one example, osteocalcin induces muscle anabolism and improves skeletal muscle function during exercise [8,9].

Both skeletal muscle and bone are major regulators of whole-body metabolism. At rest, 60–70% of the energy consumption in skeletal muscles comes from fatty acids [10,11]. Skeletal muscles also have important roles in glucose disposal by accounting for 80% of insulin-stimulated glucose uptake [12]. Bones act as an important supply of calcium to secure proper functioning of skeletal muscles and nerves [13], while skeletal muscles account for 80% of the carbohydrate storage [14]. On the other hand, bone has been shown to play a role in regulation of energy metabolism through secretion of osteocalcin [15]. Osteocalcin is a small protein (49 amino acids) that is produced by mature osteoblasts and has hormone-like properties, as it plays a role in regulation of insulin sensitivity by increasing the release of insulin from beta cells, as well as triggering the release of adiponectin from fat cells, leading to increased insulin sensitivity [15].

While the mechanical crosstalk between bone and skeletal muscle is extensively studied, the effects of myokines and osteokines on energy metabolism in osteoblastic cells and myotubes, respectively, have remained largely unknown. The aim of this study was to explore metabolic effects of conditioned medium from osteoblastic cells on myotubes and vice versa, to further elucidate the biochemical crosstalk between skeletal muscle cells and osteoblasts.

## 2. Materials and Methods

### 2.1. Materials

Dulbecco’s Modified Eagle’s Medium (DMEM)-Glutamax™ low glucose (5.5 mM), minimum Essential Medium (MEM) with L-glutamine, Dulbecco’s Phosphate Buffered Saline (DPBS; without Ca^2+^ and Mg^2+^), heat-inactivated fetal bovine serum (FBS), penicillin-streptomycin (10,000 IE/mL), amphotericin B, human epidermal growth factor (hEGF), Pierce BCA Protein Assay Kit, Power SYBR Green PCR Master Mix, Invitrogen™ nuclease-free water (1905023), High-Capacity cDNA Reverse Transcription Kit, MicroAmp^®^ Optical 96-well Reaction Plate, Nunc™ culture flasks, and MicroAmp^®^ Optical Adhesive Film were purchased from Thermo Fisher Scientific (Waltham, MA, USA). Insulin (Actrapid^®^ Penfill^®^ 100 IE/mL) was from NovoNordisk (Bagsvaerd, Denmark). Trypsin was purchased from Promega (Madison, WI, USA). HEPES, bovine serum albumin (BSA), L-ascorbic acid, β-glycerophosphate, dexamethasone, 1,25-dihydroxy vitamin D_3,_ gentamicin, β-mercaptoethanol, L-carnitine, D-glucose, oleic acid (18:1, n-9), and perchloric acid were purchased from Sigma-Aldrich (St. Louis, MO, USA). D-[^14^C(U)]glucose, [1-^14^C]oleic acid, and [^14^C]leucine were from PerkinElmer NEN^®^ (Boston, MA, USA). Ultima Gold™ XR, Pico Prias 6 mL PE vials, 96-well Isoplate^®^, UniFilter^®^-96 GF/B microplates, and TopSeal^®^-A transparent film were purchased from PerkinElmer (Waltham, MA, USA). QIA shredder and RNeasy Mini Kit were from Qiagen (Venlo, The Netherlands). Corning CellBIND^®^ tissue culturing plates were from Corning (Schiphol-Rijk, The Netherlands). Bio-Rad Protein Assay Dye Reagent Concentrate, Bio-Rad Precision Plus Protein™ Dual Color standard, Trans-Blot^®^ Turbo™ Mini-size 0.2 µm Nitrocellulose Transfer kit (#1704270), sodium dodecyl sulfate (SDS), and Tween 20 were from Bio-Rad (Copenhagen, Denmark). Odyssey^®^ Blocking Buffer (TBS) (927-50000), NewBlot™ IR stripping buffer (5×), Chameleon TM Duo Prestained Protein Ladder (928-60000), IRDye^®^ 680/800 RD donkey anti-mouse IgG secondary antibody, and IRDye^®^ 680/800 RD donkey anti-rabbit IgG secondary antibody were purchased from LI-COR Biosciences (Cambridge, UK). NuPAGE 4–12% gels and NuPAGE SDS running buffer were purchased from Life Technologies, Paisley, UK. Thin-layer chromatography silica gel 60 sheets and acetone were from Merck (Darmstadt, Germany). Free fatty acids (FFA, 2 mg/mL); cholesterol ester (CE, 2 mg/mL); and mono-, di-, triglyceride mix (4 mg/mL) were from Supelco (Bellefonte, PA, USA). Recombinant human SPARC was purchased from R&D Systems (Minneapolis, MN, USA). Rabbit anti-human phospho-Akt (Ser473) antibody (9271S), rabbit anti-human total Akt (9272) antibody, rabbit anti-human phospho-AMPKα (Thr172) antibody (2531), and rabbit anti-human total AMPKα antibody (2532) were purchased from Cell Signaling Technology^®^ Inc (Beverly, MA, US). Mouse anti-human GAPDH (sc-47724) was from Santa Cruz Biotechnology (Dallas, TX, USA).

### 2.2. Ethical Approvals

Human muscle biopsies were obtained after informed written consent and approval by the Regional Committees for Medical and Health Research Ethics (REK) North (ref. no. 2011/882) and REK South East (ref. no. 11959), Norway. The studies were conducted according to the principles outlined by the Declaration of Helsinki. Ethical approval for biopsies used for the proteomic analysis of conditioned medium from skeletal muscle cells is described in Mengeste et al. [16].

### 2.3. Cell Culturing and Harvesting of Conditioned Media

Skeletal muscle cells: Human satellite cells were established from biopsies of *Musculus vastus lateralis*, and a cell bank was established as described previously [17]. The donors used in the present study were five males and one female, 50.4 ± 10 years, with body mass index (BMI) 25 ± 14.8 kg/m^2^. Not all donors were used in all experiments. In addition, the myotube secretome data derived from [16] are from seven young male subjects, age 23.4 ± 0.9 years, with a mean BMI 23.8 ± 0.8. Cells were cultured in DMEM-Glutamax™ medium supplemented with 10% FBS, 10 ng/mL hEGF, 0.39 μg/mL dexamethasone, 0.05% BSA, 25 mM HEPES, 50 ng/mL gentamycin, 25 IU penicillin, 25 μg/mL streptomycin, and 1.25 μg/mL amphotericin B in a humidified 5% CO_2_ atmosphere at 37 °C, and the medium was changed every 2–3 days. At approximately 80% confluence, the medium was changed to myotube differentiation medium (DMEM-Glutamax™ supplemented with 2% FBS, 25 pM insulin, 25 mM HEPES, 1.25 μg/mL amphotericin B, 50 ng/mL gentamicin, 25 IU penicillin, and 25 μg/mL streptomycin) to initiate differentiation into multinucleated myotubes. In all experiments, cells were differentiated for 7 days, and the medium was changed every 2–3 days. Experiments were performed on cells from passages 3 or 4. Conditioned media (CMs) were collected and centrifuged at 100× *g* for 5 min at 4 °C. The supernatants were frozen at −20 °C and later used as reagents (SKM-CM).

For most experiments, myotubes were incubated during the last two days of culturing, i.e., after the last medium change, with an equal volume of myotube differentiation medium and CM from osteoblastic cells differentiated for 14 days (OB-CM) or an equal volume of myotube differentiation medium and fresh osteoblast induction medium (Ctr). For insulin sensitivity assay, myotubes were incubated with 0–100 nM insulin on day 7 for 15 min before harvesting. In separate experiments, recombinant human SPARC (8 µg/mL) was added to fresh myotube differentiation medium during the last two days of culturing.

Osteoblastic cells: Human bone marrow-derived mesenchymal stromal stem cells (hBMSCs) overexpressing the human telomerase reverse transcriptase (TERT) (hBMSC-TERT) were cultured in basal medium (Minimal Essential Media (MEM) with L-glutamine, 10% FBS, 1% penicillin (100 U/mL), and streptomycin (100 µg/mL)), as previously described [18]. The cells were seeded and, at 80% confluence, differentiated using osteoblastic induction medium (OBIM) containing basal medium supplemented with 10 mM β-glycerophosphate, 50 µg/mL L-ascorbic acid, 10 nM dexamethasone, and 10 nM 1,25-dihydroxy vitamin D_3_ for 14 days. The induction medium was renewed every 3–4 days. CM from 10–14 days was collected and centrifuged at 100× *g* for 5 min at 4 °C. The supernatants were frozen at −20 °C and later used as a reagent (OB-CM).

For experiments, osteoblastic cells were incubated with an equal volume of fresh OBIM and CM from differentiated myotubes (SKM-CM) or an equal volume of fresh OBIM and myotube differentiation medium (Ctr) during the last four days of culturing.

### 2.4. Total Protein Harvesting and Immunoblotting

Myotubes were washed with PBS before total protein harvesting in lysis buffer (100 mM sodium citrate, 1 mM disodium-EDTA, 1% n-octyl-β-D-glucopyranoside, pH 5.8). Cell lysates were frozen (−80 °C) and thawed (30 °C) three times before centrifugation at 10,000× *g* for 5 min and frozen at −20 °C or directly analyzed. Total protein concentrations in cell lysates were determined by measuring absorbance at 595 nm in a microplate reader (VICTOR™ X4 Multilabel Plate Reader, PerkinElmer, Waltham, MA, USA), according to the manufacturer (BioRad Laboratories, Hercules, CA, USA). BSA (0–400 µg/mL) was used to generate a standard curve for the calculation of total protein concentrations. All measurements were performed in triplicates.

Gel electrophoresis and immunoblotting of cell lysates were performed using NuPAGE 4–12% gels (Life Technologies) and supplied NuPAGE SDS running buffer, before transferring to a nitrocellulose membrane (0.2 µm) in a Trans-Blot^®^ Turbo™ Transfer System (Bio-Rad, Copenhagen, Denmark). Blotting was performed using supplied 1× transfer buffer. Ponceau staining was used to verify protein transfer. Membranes were blocked with Intercept Blocking Buffer (Tris-buffered saline, TBS; LI-COR, Lincoln, NE, USA) for 1 h, prior to incubation overnight at 4 °C with primary antibody: Rabbit anti-human phospho-Akt (Ser473) (1:1000), rabbit anti-human total Akt (1:1000), rabbit anti-human phospho-AMPKα (Thr172) (1:1000), rabbit anti-human total AMPKα (1:1000), or mouse anti-human GAPDH (1:10,000) overnight at 4 °C. Membranes were then washed 3–4 times in 0.2% T-TBS (Tris-buffered saline with Tween 20) and probed with donkey anti-mouse IR dye 680LT or 800CW (1:10,000;) or donkey anti-rabbit 680LT or 800CW (1:10,000) for 1 h at room temperature. Following a washing procedure, membranes were quickly dried and scanned using the Odyssey-CLx Imaging System (LI-COR, Lincoln, NE, USA). Band intensities were quantified with Image Studio Lite (version 5.2) (LI-COR, Lincoln, NE, USA). Akt phosphorylation was normalized versus total Akt expression, while AMPKα phosphorylation was normalized versus total AMPKα expression.

### 2.5. RNA Isolation and qPCR

Total RNA from myotubes was isolated using the Qiagen RNeasy Mini kit, following the manufacturer’s protocol. Nanodrop ND-1000 (Thermo Scientific, Wilmington, DE, USA) was used to quantify RNA concentrations. RNA was reversely transcribed with High-Capacity cDNA Reverse Transcription using a PerkinElmer 2720 Thermal Cycler (PerkinElmer, Waltham, MA, USA). To perform quantitative PCR, SYBR™ Green Master Mix was used, and quantification of target genes was carried out in duplicates using 30 µM of each primer. The assay was run in StepOnePlus™ (Applied Biosystems, Waltham, MA, USA). The mRNA expressions were normalized to the housekeeping controls *RPLP0* and/or *GAPDH*. Primers (Table 1) were designed using Primer Express version 2.0^®^ (Applied Biosystems, Waltham, MA, USA).

### 2.6. Acute Substrate Oxidation Assay

Differentiated myotubes (day 7) or osteoblastic cells (day 14) were cultured in 96-well plates, and radiolabeled substrate, D-[^14^C(U)]glucose (18.5 kBq/mL, 200 μM) or [1-^14^C]oleic acid (18.5 kBq/mL, 100 μM), was added acutely during 4 h of CO_2_ trapping, as described previously [19]. CO_2_ production (complete oxidation) and cell-associated radioactivity (CA) were assessed using a PerkinElmer 2450 MicroBeta^2^ scintillation counter (PerkinElmer, Waltham, MA, USA). Total substrate uptake was then calculated as CO_2_ + CA. Data were normalized versus total protein content in each well, which was determined by the Bio-Rad protein assay using a VICTOR™ X4 Multilabel Plate Reader (PerkinElmer, Waltham, MA, USA).

### 2.7. Lipid Distribution

Myotubes were incubated with [1-^14^C]oleic acid (18.5 kBq/mL, 100 μM) for 4 h. After incubation, the cells were washed twice with PBS and harvested in 350 μL 0.1% SDS per well. Lipids were extracted using chloroform–methanol (2:1, *v*/*v*, Folch extraction) and 0.9% sodium chloride solution pH 2 [20]. The organic phase was evaporated by N_2_, and extracted lipids were redissolved in 130 µL hexane and separated by thin-layer chromatography. A non-polar solvent mixture of hexane:diethyl ether:acetic acid (65:35:1) was used to separate the lipids. The lipid bands were visualized using iodine, and quantification of radioactivity was assessed by liquid scintillation (Packard Tri-Carb 1900 TR, PerkinElmer, Waltham, MA, USA). The amount of neutral and polar lipids was related to the total protein concentration determined by the Pierce BCA Protein Assay Kit, as described by the supplier.

### 2.8. Protein Synthesis by Leucine Incorporation

Myotubes were incubated with [^14^C]leucine (37 kBq/mL, 0.8 mM) in differentiation medium for 24 h. The cells were then washed with PBS and lysed in 0.01% SDS. The amount of total protein in cell lysates was determined by the Pierce BCA Protein Assay Kit. Total proteins were precipitated with 1% BSA in 50% trichloroacetic acid overnight at −20 °C. The next day, the cell lysates were centrifuged (12,000× *g*) to form a protein pellet. The pellet was washed in acetone before centrifugation, airdried, and resuspended in SDS-NaOH. Radioactivity was measured by liquid scintillation (Packard Tri-Carb 1900 TR, PerkinElmer, Waltham, MA, USA), and the amount of labeled protein was normalized to the total protein content.

### 2.9. Proteomic Analysis

For secretome analysis, the cells were washed with PBS and incubated with serum-free media for 24 h before CM collection. For analysis of cell lysates, the cells were washed twice with PBS, harvested in PBS, centrifuged at 160× *g* for 5 min at 4 °C, and the cell pellet frozen at −80 °C. The cell pellet was thawed, dissolved in RIPA buffer, and sonicated for 2 × 30 s with intervals of 30 s. Cell debris was removed by centrifugation (14,000× *g*) for 10 min. The total protein concentration was determined by the Pierce BCA Protein Assay Kit, and for each replicate, the equal amount (10 µg) of total protein was precipitated on amine beads, as previously described [21]. CM samples for secretome analysis were concentrated with 10 kD cutoff filter by spinning at 3000 g for 30 min. The precipitated proteins on beads and concentrated secretome samples were dissolved in 50 mM ammonium bicarbonate, reduced, alkylated, and digested with trypsin (1:50 enzyme:protein ratio) at 37 °C overnight. Digested peptides were acidified, and the peptides were loaded to Evosep C18 tips.

LC-MS/MS analysis of the whole cell lysates was carried out using an EvosepOne LC system (Evosep, Odense, Denmark) coupled to a timsTOF fleX mass spectrometer (Bruker Daltonics, Billerica, MA, USA), and for the secretomes using EvosepOne, coupled to a QExactive HF mass spectrometer (Thermo). Peptide digest from cell lysate (200 ng) or 20 uL of peptide digest from secretomes was loaded on a capillary C18 Evosep column (15 cm length, 150 μm inner diameter, 1.5 μm particle size, 120; Evosep), and peptides were separated using a 44 min gradient. Raw files from LC-MS/MS analyses were submitted to MaxQuant 2.0.3.0 software for protein identification and label-free quantification. Parameters were set as follows: Carbamidomethyl (C) was set as a fixed modification and protein N-acetylation and methionine oxidation as variable modifications. The first search error window was 20 ppm, and the mains search error was 6 ppm. Trypsin without the proline restriction enzyme option was used, with two allowed miscleavages. Minimal unique peptides were set to one, and the FDR allowed was 0.01 (1%) for peptide and protein identification. The Uniprot human database was used. Generation of reversed sequences was selected to assign the FDR rate. Perseus version 1.6.15.0 was used for further analysis of MaxQuant data. Intensity-based absolute quantification (iBAQ) intensity was used to determine the abundance of secreted proteins, and protein detection in three of four OB-CM samples and five of seven SKM-CM samples are listed. A paired two-tailed Student’s *t*-test (*p* < 0.05) was performed to determine differences in protein expression between myotubes stimulated with OB-CM and control myotubes (Ctr). STRING version 11.5 (https://string-db.org/, accessed on 7 June 2023) was used to group differentially regulated protein into protein classes and Gene Ontology (GO) Biological Process annotations for the conditioned media, while KEGG and Reactome annotations were used for myotube lysate proteome analysis. The human MitoCarta 3.0 [22] was used to identify proteins related to mitochondrial function from our list of significantly regulated proteins. The mass spectrometry proteomics data were deposited in the ProteomeXchange Consortium via the PRIDE [23] partner repository with the dataset identifiers PXD045723 (myotube lysates and OB-CM) and PXD033025 (SKM-CM).

### 2.10. Statistics

All values are reported as mean ± SEM. The value *n* represents the number of individual experiments, each with replicate measurements. Statistical analyses were performed using GraphPad Prism 9.3.1 for Windows (GraphPad Software Inc., La Jolla, CA, USA). Unpaired *t*-tests or paired *t*-tests were performed when appropriate. A *p*-value < 0.05 was considered significant.

## 3. Results

### 3.1. Conditioned Medium from Differentiated Osteoblastic Cells Increases Glucose Metabolism in Human Skeletal Muscle Cells

**Figure 1:**
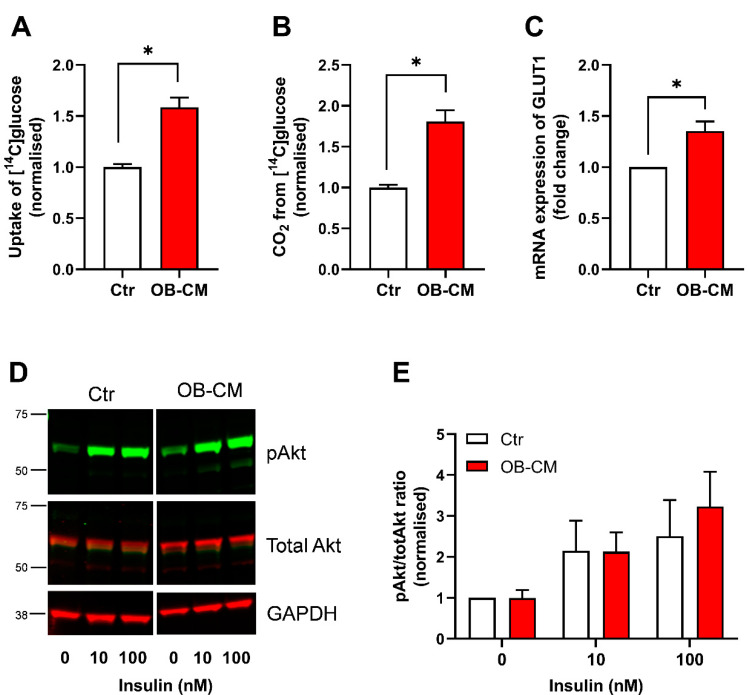
Effects of conditioned medium from differentiated osteoblastic cells (OB-CM) on glucose metabolism and insulin response in myotubes. Human myoblasts were differentiated to myotubes for 7 days and incubated the last 2 days with 1:1 of myotube differentiation medium and conditioned medium from osteoblastic cells differentiated for 14 days (OB-CM) or 1:1 of myotube differentiation medium and osteoblast induction medium (Ctr). (**A**,**B**) Oxidation (trapped CO_2_) and cell-associated radioactivity (CA) of [^14^C]glucose were measured after incubation for 4 h and normalized to Ctr (9 independent experiments, with 4–8 biological replicates in each experiment). Results are shown as mean ± SEM. * *p* < 0.05 vs. Ctr, unpaired *t*-test. (**A**) Cellular uptake of glucose (CO_2_ + CA). (**B**) Complete oxidation of glucose. (**C**) Relative mRNA expression of glucose transporter 1 (GLUT1/SLC2A1) correlated to the housekeeping GAPDH and normalized to Ctr (5 independent experiments). * *p* < 0.05 vs. Ctr, paired *t*-test. (**D**,**E**) On the last day of culturing, myotubes were incubated for 15 min with or without 10 or 100 nM insulin before the cell lysates were harvested and assessed by immunoblotting. (**D**) A representative immunoblot showing pAkt (upper panel, green), total Akt (middle panel, red), and the housekeeping GAPDH (lower panel, red). (**E**) Quantified immunoblots from 3 individual experiments in D presented as relative to Ctr. Absolute values for Ctr: (**A**) 29.3 ± 4.0 nmol/mg protein, (**B**) 24.7 ± 3.1 nmol/mg protein.

To study the effects of osteokines on energy metabolism in human skeletal muscle cells, myotubes were treated with conditioned medium from differentiated osteoblastic cells (OB-CM) the two last days of culturing (days 5–7) before incubation with radiolabeled substrates for 4 h. First, the effects of OB-CM on glucose metabolism in myotubes using [^14^C]glucose were investigated. An increase in both glucose uptake and oxidation was observed in myotubes treated with OB-CM compared to non-conditioned cells (control) (Figure 1A,B). In line with the improved glucose uptake, increased mRNA expression of the glucose transporter 1 (GLUT1/SLC2A1) was also observed in myotubes treated with OB-CM (Figure 1C). To analyze the effects of OB-CM on insulin sensitivity, basal and insulin-stimulated Akt phosphorylation were investigated. Increasing insulin concentrations tended to enhance the pAkt/total Akt ratio; however, no effect of treatment with OB-CM was observed (Figure 1D,E).

### 3.2. Conditioned Medium from Differentiated Osteoblastic Cells Reduces Oleic Acid Metabolism in Human Skeletal Muscle Cells

**Figure 2:**
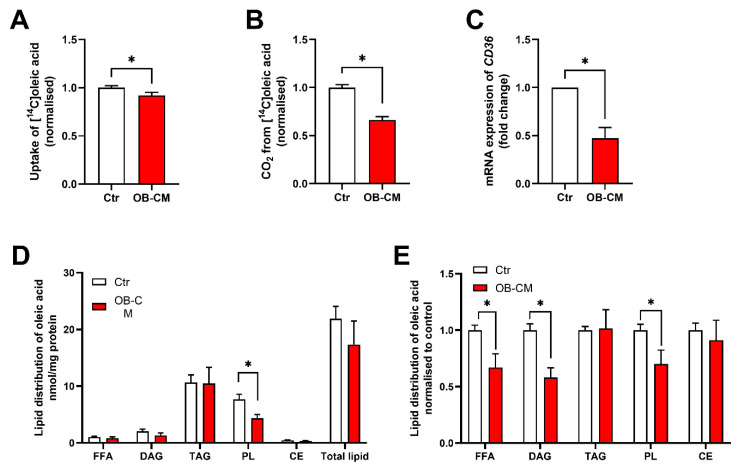
Effects of conditioned medium from differentiated osteoblastic cells (OB-CM) on fatty acid metabolism in myotubes. Human myoblasts were differentiated to myotubes for 7 days and incubated the last 2 days with an equal volume of myotube differentiation medium and conditioned medium from osteoblastic cells (OB-CM) differentiated for 14 days or an equal volume of myotube differentiation medium and osteoblast induction medium (Ctr). (**A**,**B**) Oxidation (trapped CO_2_) and cell-associated radioactivity (CA) of [^14^C]oleic acid were measured after incubation for 4 h and normalized to Ctr (9 independent experiments, with 4–8 biological replicates in each experiment). Results are shown as mean ± SEM. * *p* < 0.05 vs. Ctr, unpaired *t*-test. (**A**) Cellular uptake of oleic acid (CA + CO_2_). (**B**) Complete oxidation of oleic acid. (**C**) Relative mRNA expression of the fatty acid transporter CD36 correlated to the housekeeping GAPDH and normalized to Ctr (5 independent experiments). Results are shown as mean ± SEM. * *p* < 0.05 vs. Ctr, paired *t*-test. (**D**,**E**) Myotubes were incubated with 100 μM [^14^C]oleic acid for 4 h. Lipids were separated by thin-layer chromatography and quantified by liquid scintillation (3 independent experiments, with 3 replicates in each experiment). Data are shown as mean ± SEM. * *p* < 0.05 vs. Ctr, unpaired *t*-test. (**D**) Absolute values for lipid distribution in nmol/mg protein. (**E**) Lipid distribution as percent of Ctr. CE, cholesteryl ester; DAG, diacylglycerol; FFA, free fatty acid; PL, phospholipid; TAG, triacylglycerol. Absolute values for Ctr: (**A**) 118.6 ± 15.9 nmol/mg protein, (**B**) 18.9 ± 2.1 nmol/mg protein.

Next, the effects of OB-CM on fatty acid metabolism were investigated using [^14^C]oleic acid for 4 h. Myotubes incubated with OB-CM showed reduced oleic acid uptake and oxidation (Figure 2A,B), as well as a reduced mRNA expression of the fatty acid transporter CD36 (Figure 2C). To further investigate changes in oleic acid metabolism, fatty acid distribution to complex lipids was examined. The myotubes were incubated with [^14^C]oleic acid for 4 h, and incorporation of oleic acid into different lipid classes was measured by thin-layer chromatography. OB-CM significantly reduced incorporation of oleic acid into complex lipids, such as diacylglycerol (DAG) and phospholipid (PL), compared to the control (Figure 2D,E).

### 3.3. Conditioned Medium from Differentiated Myotubes Increases Glucose Uptake and Reduces Oleic Acid Uptake in Osteoblastic Cells

**Figure 3:**
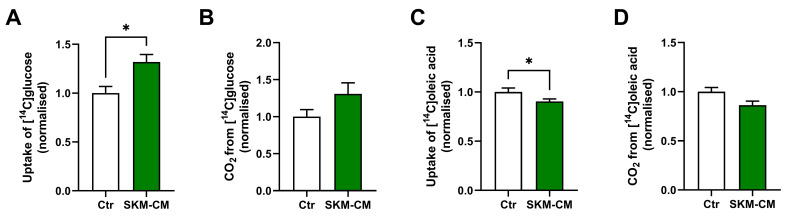
Effects of conditioned medium from differentiated myotubes (SKM-CM) on glucose and oleic acid metabolism in osteoblastic cells. Human BMSC-TERT cells were differentiated to osteoblastic cells for 14 days and incubated for the last 4 days with an equal volume of osteoblast differentiation medium and conditioned medium from 7 days-differentiated human skeletal myotubes (SKM-CM) or an equal volume of osteoblast differentiation medium and myotube differentiation medium (Ctr). Oxidation (trapped CO_2_) and cell-associated radioactivity (CA) of [^14^C]glucose or [^14^C]oleic acid were measured after incubation for 4 h and normalized to Ctr (3 independent experiments, with 4 biological replicates from each experiment). (**A**) Cellular uptake of glucose (CO_2_ + CA). (**B**) Complete oxidation of glucose. (**C**) Cellular uptake of oleic acid (CA + CO_2_). (**D**) Complete oxidation of oleic acid. Data are shown as mean ± SEM. * *p* < 0.05 vs. Ctr, unpaired *t*-test. Absolute values for Ctr: (**A**) 22.8 ± 1.9 nmol/mg protein, (**B**) 8.7 ± 1.1 nmol/mg protein, (**C**) 35.7 ± 2.3 nmol/mg protein, (**D**) 10.0 ± 0.7 nmol/mg protein.

We were further interested in studying the effects of conditioned medium from differentiated myotubes (SKM-CM) on osteoblastic cells. Human BMSC-TERT cells were differentiated to osteoblastic cells for 14 days and treated with SKM-CM during the last 4 days of culturing (days 10–14) before incubation with [^14^C]glucose or [^14^C]oleic acid for 4 h. Glucose uptake was increased in the osteoblastic cells, while glucose oxidation tended to increase (Figure 3A,B). In contrast, uptake of oleic acid was reduced in differentiated osteoblastic cells, but fatty acid oxidation was not affected by SKM-CM treatment (Figure 3C,D).

### 3.4. Secretome Analysis of Conditioned Medium from Differentiated Osteoblastic Cells and Myotubes

Since conditioned medium from differentiated osteoblastic cells and myotubes showed pronounced effects on glucose and fatty acid metabolism in myotubes and osteoblastic cells, respectively, we wanted to identify the present proteins in the OB-CM and SKM-CM by proteomic analysis. The proteomic data of SKM-CM from control cells were obtained from the analysis recently published by Mengeste et al. [16] (ProteomeXchange Consortium, dataset identifier PXD033025). The present analysis detected 284 proteins in the OB-CM (Supplementary Table S1) and 762 proteins in the SKM-CM (Supplementary Table S2). In total, 215 proteins were present in both conditioned media. The 20 most abundant proteins in OB-CM and SKM-CM are shown in Table 2 and Table 3, respectively, and classified based on the Biological Process of Gene Ontology (GO). Several of the most abundant proteins in both conditioned media were classified in the GO-terms: cytoskeleton organization, regulation of cellular component organization, organelle organization, and regulation of multi-cellular organismal process. In OB-CM, 10 of the 20 most abundant proteins were classified by the GO term Regulated exocytosis (Table 2), while 9 of the 20 most abundant proteins in SKM-CM were classified by the term Response to cytokine (Table 3). The 5 common proteins found between the 20 most abundant proteins in both conditioned media were ACTG1 (actin, cytoplasmic 2), VIM (vimentin), SPARC (secreted protein acidic and protein rich), LGALS1 (galectin-1), and PFN1 (profilin-1) (Table 2 and Table 3).

### 3.5. SPARC Increases Oleic Acid Oxidation in Human Skeletal Muscle Cells

**Figure 4:**
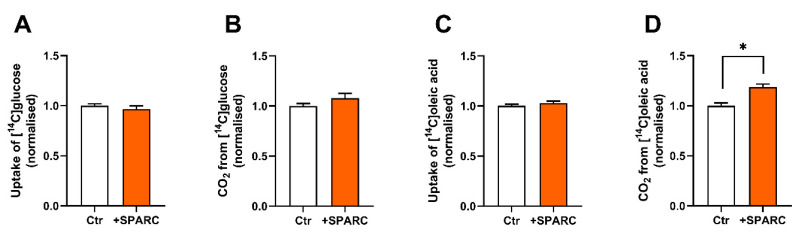
Effect of recombinant human SPARC on glucose and fatty acid metabolism in myotubes. Human myoblasts were differentiated to myotubes for 7 days and incubated the last 2 days with or without recombinant human SPARC (8 µg/mL). Oxidation (trapped CO_2_) and cell-associated radioactivity (CA) of [^14^C]glucose or [^14^C]oleic acid was measured after incubation for 4 h and normalized to Ctr (3 independent experiments, with 4 biological replicates from each experiment). (**A**) Cellular uptake of glucose (CO_2_ + CA). (**B**) Complete oxidation of glucose. (**C**) Cellular uptake of oleic acid (CA + CO_2_). (**D**) Complete oxidation of oleic acid. Data are shown as mean ± SEM. * *p* < 0.05 vs. Ctr, unpaired *t*-test. Absolute values for Ctr: (**A**) 42.1 ± 4.5 nmol/mg protein, (**B**) 24.7 ± 3.3 nmol/mg protein, (**C**) 105.6 ± 2.8 nmol/mg protein, (**D**) 18.0 ± 1.8 nmol/mg protein.

SPARC was detected as one of the most abundant proteins secreted from both osteoblastic cells and myotubes. Since SPARC previously has been shown to mediate myotube differentiation and formation [24,25,26], it was selected for further studies as a possible contributor to the observed metabolic effects of OB-CM on myotubes. In contrast to the decrease observed in treatment with OB-CM (Figure 3C), incubation with 8 µg/mL SPARC for two days increased fatty acid oxidation in the myotubes compared to the control, while no effects on glucose uptake nor oxidation were observed (Figure 4).

### 3.6. Conditioned Medium from Differentiated Osteoblastic Cells Inhibits PGC1α mRNA Expression and Protein Synthesis in Human Skeletal Muscle Cells

**Figure 5:**
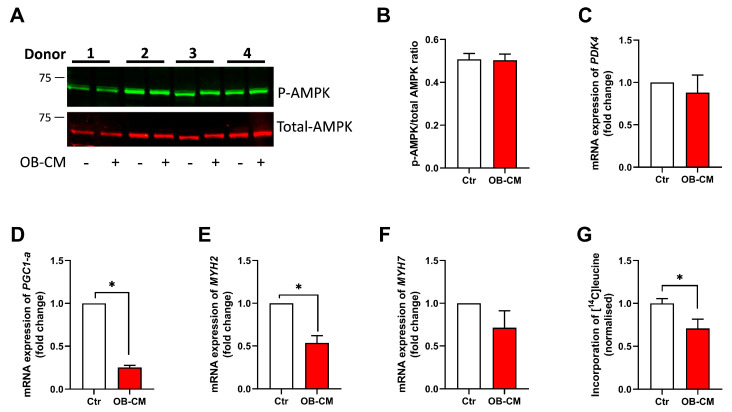
Effects of conditioned medium from osteoblastic cells (OB-CM) on regulation of selected markers of energy metabolism and myotube fiber types. Human myoblasts were differentiated to myotubes for 7 days and incubated the last 2 days with an equal volume of myotube differentiation medium and conditioned medium from osteoblastic cells (OB-CM) differentiated for 14 days or an equal volume of myotube differentiation medium and osteoblast induction medium (Ctr). (**A**) One representative immunoblot from 5 independent experiments showing phosphorylated AMPKα (p-AMPK) (upper panel, green) and total AMPKα (lower panel, red). (**B**) Quantified immunoblots of p-AMPKα versus total AMPKα of 4 out of 5 independent experiments using different donors shown in A. (**C**–**F**) Relative mRNA expressions of pyruvate dehydrogenase kinase (*PDK*; (**C**)) 4, peroxisome proliferator-activated receptor gamma coactivator 1-alpha (*PGC1α*; (**D**)), myosin heavy chain (*MYH*; (**E**)) *2*, and *MYH7* (**F**) correlated to the housekeeping *GAPDH* and normalized to Ctr. Results are shown as mean ± SEM (*n* = 5 donors). * *p* < 0.05 vs. Ctr, paired *t*-test. (**G**) After 7 days of differentiation, protein synthesis was measured by incubating myotubes with [^14^C]leucine (800 µM) for 24 h before cell-associated radioactivity was quantified by liquid scintillation (4 independent experiments, with 3 biological replicates from each experiment). Data are shown as mean ± SEM and normalized to Ctr. * *p* < 0.05 vs. Ctr, unpaired *t*-test. Absolute values for Ctr in (**G**) 59.8 ± 14.8 nmol/mg protein.

As the metabolic effects of OB-CM on myotubes were more pronounced than the metabolic effects of SKM-CM on osteoblastic cells, we chose to further explore the effect of OB-CM on myotubes. AMP-activated protein kinase (AMPK) is important for cellular energy homeostasis and is activated when cellular energy is low to trigger glucose and fatty acid uptake and oxidation [27]. Due to its molecular function, we wanted to investigate whether the observed effects of OB-CM on glucose metabolism in myotubes could be explained by activation of AMPK. However, no difference in the ratio between phosphorylated and total AMPKα was observed in myotubes treated with OB-CM and control cells (Figure 5A,B).

Regulation of mRNA expression of selected markers for energy metabolism and muscle fiber types was assessed using qPCR. Myotubes incubated with OB-CM did not alter the expression of pyruvate dehydrogenase kinase 4 (*PDK4*); however, the mRNA expression of the peroxisome proliferator-activated receptor gamma coactivator 1-alpha (PGC1α) and of the muscle fiber marker myosin heavy chain 2 (*MYH2*) was significantly reduced (Figure 5C–E). No change in the mRNA expression of *MYH7* was found (Figure 5F). Next, to study whether treatment with OB-CM affected protein synthesis, differentiated myotubes were incubated with [^14^C]leucine for 24 h. The results showed a significant reduction in protein synthesis after the incubating of myotubes with OB-CM compared to the control (Figure 5G).

### 3.7. Molecular Proteomic Changes Correlate with Functional Effects of OB-CM on Myotubes Energy Metabolism

**Figure 6:**
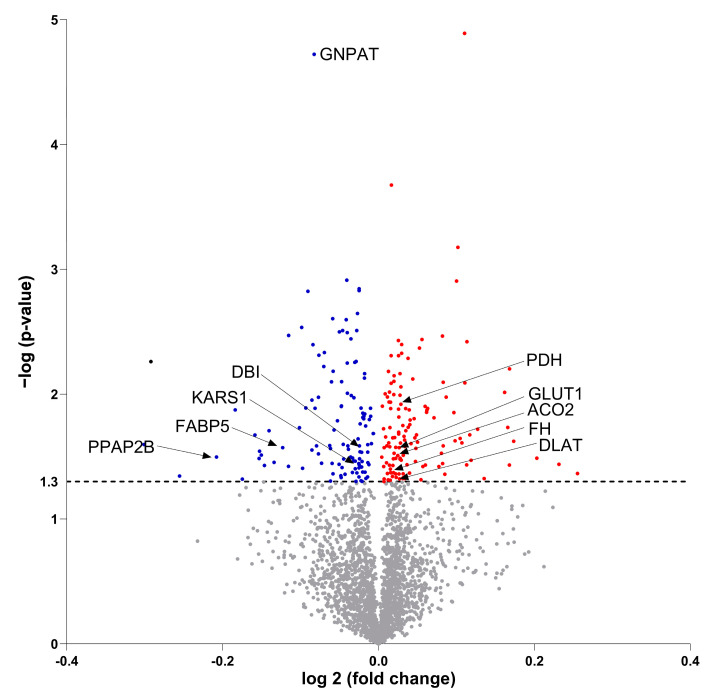
Differential protein expression in myotubes incubated with or without conditioned medium from differentiated osteoblastic cells. Human myoblasts were differentiated to myotubes for 7 days and incubated the last 2 days with an equal volume of myotube differentiation medium and conditioned medium from osteoblastic cells (OB-CM) differentiated for 14 days or an equal volume of myotube differentiation medium and osteoblast induction medium (Ctr). On day 7 of differentiation, the myotubes from four different donors in each group were harvested for proteomic analysis. Volcano plot of all regulated and identified proteins (2726) is shown, including significantly downregulated proteins (blue), significantly upregulated proteins (red), and non-significantly regulated proteins (grey). Some proteins mentioned in the body text are shown. Statistical significance was calculated as the difference between the two groups using paired *t*-test, and differences were considered significant at *p* < 0.05. ACO2, aconitase 2; DBI, acyl-CoA-binding protein; DLAT, dihydrolipoyllysine-residue acetyltransferase component of pyruvate dehydrogenase complex, mitochondrial; FABP5, fatty acid-binding protein; FH, fumarate hydratase; GLUT1, glucose transporter 1; GNPAT, dihydroxyacetone phosphate acyltransferase; KARS1, lysyl-TRNA synthetase 1; PDH, pyruvate dehydrogenase; PPAP2B, lipid phosphate phosphohydrolase 3.

To further investigate the effects of OB-CM on myotubes, proteomic analysis of myotube lysates was performed. The analysis detected 2726 proteins (Figure 6, Supplementary Table S3), of which 130 proteins were significantly upregulated (red dots) and 116 proteins were significantly downregulated (blue dots) after treatment with OB-CM compared to the control.

**Figure 7:**
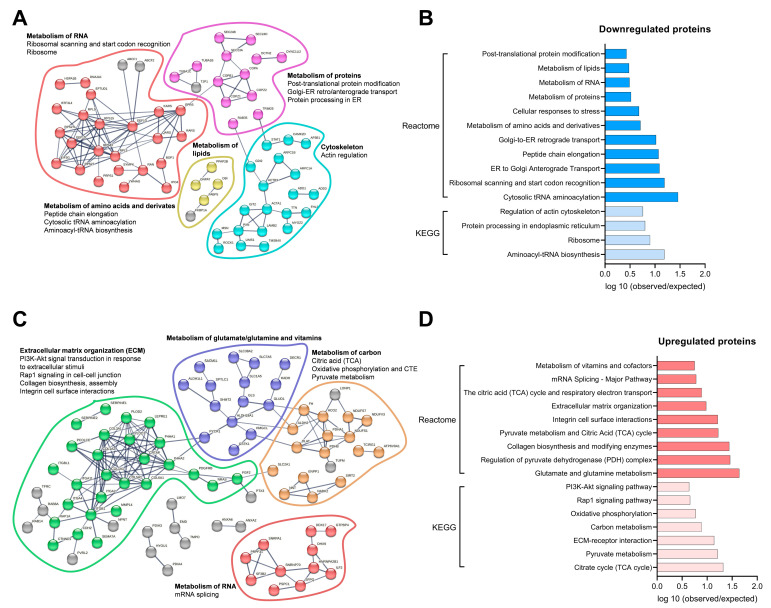
Pathway analysis of significantly regulated protein expression in myotubes incubated with or without conditioned medium from differentiated osteoblastic cells. Human myoblasts were differentiated to myotubes for 7 days and incubated the last 2 days with an equal volume of myotube differentiation medium and conditioned medium from 14 days-differentiated osteoblastic cells (OB-CM) or an equal volume of myotube differentiation medium and osteoblast induction medium (Ctr). On day 7 of differentiation, the myotubes from four different donors in each group were harvested for proteomic analysis. (**A**) Network representation of downregulated proteins with a cluster representation of involved KEGG/Reactome pathways. (**B**) Selected pathways of downregulated proteins, based on KEGG and Reactome, represented by the strength value (log 10 (observed/expected proteins) in a specific pathway). (**C**) Network representation of upregulated proteins with a cluster representation of involved KEGG/Reactome pathways. (**D**) Selected pathways of upregulated proteins, based on KEGG and Reactome, represented by the strength value (log 10 (observed/expected) protein) in a specific pathway. Results from KEGG and Reactome pathways analyses performed using STRING software, version 11.5 (https://string-db.org/, accessed on 21 June 2023).

To investigate the biological role of the down- or upregulated proteins, analyses based on KEGG and Reactome pathway databases were performed. Pathways found within the KEGG and Reactome databases were analyzed by the strength of association between observed and expected numbers of proteins in the respective pathways. With a confidence of 0.7, downregulated proteins were represented in a functional and physical protein interaction network (Figure 7A). In this network, proteins were clustered by metabolic pathways assigned by the KEGG and Reactome databases and observed in the analysis (Figure 7B). Interestingly, within all detected pathways, some downregulated proteins, such as acyl-CoA-binding protein (DBI), fatty acid-binding protein (FABP5), lipid phosphate phosphohydrolase 3 (PPAP2B), and dihydroxyacetone phosphate acyltransferase (GNPAT), are involved in lipid metabolism.

Also, upregulated proteins were represented and clustered, as described above, by assigning pathways from the KEGG and Reactome databases (Figure 7C,D). After this analysis, upregulated proteins were assigned to pathways involved in extracellular matrix organization, carbon metabolism, glutamate, vitamins, and RNA metabolism. Proteins, like glucose transporter 1 (GLUT1/SLC2A), pyruvate dehydrogenase (PDH) and mitochondrial dihydrolipoyllysine-residue acetyltransferase component of pyruvate dehydrogenase complex (DLAT), shown to be involved in glucose metabolism were upregulated. Fumarate hydratase (FH) and aconitase 2 (ACO2), involved in the tricarboxylic acid cycle (TCA), or NADH:ubiquinone oxidoreductase (complex I) (NDUF), part of the electron chain transport, were also among the upregulated proteins.

Due to the representation of mitochondrial proteins in the analyzed samples, we decided to further explore which significantly regulated proteins were actually identified and related to mitochondrial function. We, therefore, performed a comparison analysis between the significantly regulated proteins and the MitoCarta 3.0. database ([22], https://personal.broadinstitute.org/scalvo/MitoCarta3.0/human.mitocarta3.0.html, accessed on 20 June 2023), which resulted in a total of 26 upregulated and 2 downregulated proteins in common (Table 4).

## 4. Discussion

Bone and skeletal muscle are connected in the musculoskeletal system, not only by mechanical interaction, but also through biochemical crosstalk [5,28,29]. This biochemical crosstalk between bone and skeletal muscle is mediated via the secretion of mediators from one tissue (myokines from skeletal muscle and osteokines from bone cells), which are able to affect and regulate the adjacent tissue [5,28,29]. In this study, we chose to compare metabolic responses of conditioned media-treated cells with non-conditioned cells. This approach has previously been used to explore changes in, e.g., lipolysis and gene expression induced by conditioned medium given to the acceptor cells [30,31]. Another approach would be to use transwell co-cultures [32], allowing indirect cell-to-cell contact; however, due to different culturing conditions for growth and differentiation of myotubes and osteoblastic cells, this approach was difficult to implement.

By using conditioned media, we demonstrated a crosstalk between myotubes and osteoblastic cells on energy metabolism, showing that conditioned medium from osteoblastic cells (OB-CM) reduced the uptake and oxidation of oleic acid in skeletal muscle cells, whereas the uptake and oxidation of glucose were increased. In addition, mRNA expression and proteomic analysis showed that exposure of myotubes to OB-CM increased expression of proteins involved in glucose uptake, glycolytic pathways, and the TCA cycle; however, reduced expression of some proteins involved in the metabolism of lipids was observed. Similar effects on energy metabolism were observed in osteoblastic cells treated with conditioned medium from myotubes (SKM-CM), showing increased glucose uptake and reduced oleic acid uptake. The results indicate a shift in fuel preference from fatty acids to glucose metabolism in both cell types, induced by the conditioned media from the opposite cell type; however, the metabolic effects were more pronounced in myotubes.

Glucose and fatty acids are important fuels for both osteoblasts [33] and skeletal muscle cells [11,34]. Osteoblasts primarily use glucose as the main energy source [35]; however, during osteoblast differentiation, fatty acid uptake and beta-oxidation increase [36], and fatty acid oxidation has been shown to be required for bone-forming osteoblasts in bone repair and skeletal development [33]. Skeletal muscle metabolic flexibility, i.e., the ability to shift between fatty acid and glucose fuels based on fasted or fed state and the circulating levels of insulin, has been emphasized [34]. During exercise in vivo, the intensity and duration of the activity regulate the balance between skeletal muscle glucose and fatty acid metabolism (see, e.g., [37]); however, in our in vitro cell model, the myotubes are not contracting. We found no effect on insulin-induced AKT phosphorylation in myotubes after exposure to OB-CM, indicating that insulin sensitivity was not affected. In line with the reduced oleic acid uptake, incorporation of oleic acid into complex lipids, such as DAG and PL in myotubes exposed to OB-CM, was reduced. Possible clinical applications of our current finding of a shift in fuel preference from fatty acid to glucose metabolism are not clear; however, non-insulin-dependent pathways to increase glucose uptake and utilization might be beneficial in, e.g., conditions with insulin resistance.

To study secreted proteins from either osteoblastic cells or myotubes, we performed proteome analyses of the secretome from both cell types. A total of 284 and 762 proteins were identified in the condition medium from differentiated osteoblastic cells and myotubes, respectively. Surprisingly, some of the most known myokines and osteokines, such as irisin, interleukin 6 (IL-6), fibroblast growth factor 2 (FGF-2), insulin growth factor 1 (IGF-1), myostatin (i.e., myokine) and osteocalcin (OCN), RANK ligand (RANKL), and FGF-23 (i.e., osteokine), were not detected in our samples (for review, see [5,28,29]), possibly indicating that the numbers of secreted proteins may vary under different conditions. Exercise-induced myokines, also known as exerkines [5], are most studied as secreted proteins from skeletal muscle cells. However, in our cell model, myotubes are not contracting. Another explanation could be that proteomic analyses do not detect every secreted protein due to the protocols used to isolate, detect, and identify proteins. In addition, the secretome will contain other active substances that are not proteins (e.g., lactate and prostaglandins), which were not analyzed in this study.

SPARC, also known as osteonectin, was one of the most abundant proteins in the secretome from osteoblastic cells. In mouse C2C12 cells, stimulation with SPARC was shown to increase myoblast differentiation, myogenin and mitochondrial proteins expression, as well as AMPK phosphorylation, indicating a role of SPARC in the proliferation, differentiation, and metabolism of skeletal muscle cells [24,25]. Our results showed increased oleic acid oxidation when primary human myotubes were incubated with SPARC, in contrast to treatment with OB-CM, indicating that SPARC is not the osteokine responsible for the reduction in oleic acid metabolism observed after treatment with OB-CM. A wide range of proteins were measured in the conditioned media. It is not likely that only one single protein is responsible for the observed effects, but a combination of several mediators in the secretome, including non-protein substances. Interestingly, SPARC has also been described as a myokine [38]. Previously, it has been shown that expression of secreted SPARC was increased in human myotubes during differentiation [39]. In line with this, we found herein a high abundance of SPARC in SKM-CM using proteome analysis, suggesting that SPARC is both an osteokine and a myokine.

While OB-CM affected both glucose and oleic acid uptake and oxidation in myotubes, only uptake of these substrates was affected by SKM-CM in osteoblastic cells. It is interesting, however, that the changes in the uptake of glucose and oleic acid were regulated in the same direction in both cell types. Of the 284 proteins found in OB-CM, 215 were also detected in SKM-CM; thus, a more general pattern in communication between these tissues may be present.

In myotubes exposed to OB-CM, proteomic analysis of cell lysate revealed 130 significantly upregulated and 116 downregulated proteins. Among the downregulated proteins were acyl-CoA-binding protein (DBI), fatty acid-binding protein (FABP5), lipid phosphate phosphohydrolase 3 (PPAP2B), and dihydroxyacetone phosphate acyltransferase (GNPAT), all involved in lipid metabolism. DBI, PPAP2B, and FABP5 are all lipid transporters from either external or internal cell membranes [40,41,42], and together with the decreased mRNA expression of CD36 in myotubes after incubation with OB-CM, these data support the decrease in oleic acid uptake and storage and the decreased oxidation observed in the functional experiments. Also, proteins involved in amino acid and protein metabolism were downregulated, which could explain the reduction of leucin incorporation observed in myotubes cultured in OB-CM compared to control cells. Both mRNA and protein expression of the glucose transporter GLUT1 were increased, in line with the increased glucose uptake after exposure to OB-CM. Other upregulated proteins were related to pathways involved in extracellular matrix organization, carbon metabolism, glutamate, vitamins, and RNA metabolism.

Around 20% of the proteins upregulated after exposure to OB-CM were associated with the mitochondria, supporting that OB-CM regulated mitochondrial function via carbohydrates in myotubes, as observed by increased glucose oxidation. Only two mitochondria-related proteins were downregulated and involved in fatty acid transport (DBI) and mitochondrial tRNA amino acid biosynthesis (lysyl-tRNA synthetase 1, KARS1) [43]. In addition, PGC1α mRNA expression was reduced, indicating a reduced mitochondrial biogenesis [44], despite increased expression of several mitochondrial proteins.

Human skeletal muscle expresses different isoforms of myosin heavy chain. The major myosin heavy chain isoforms are classified as fast (MyHC-2A, MyHC-2x; respective genes *MYH2* and *MYH1*) and slow (MyHC-β; gene *MYH7*) [45]. An increased expression of fast isoforms would provide a more glycolytic phenotype with reduced fatty acid metabolism, in line with our findings. However, after exposure of myotubes to OB-CM, we found reduced MYH2 mRNA expression and no changes in fiber types in the proteomic analysis; thus, a change in fiber type may not be the explanation for the changes in fuel preference in our study.

In conclusion, treatment with conditioned medium from differentiated osteoblastic cells increased glucose metabolism and reduced oleic acid metabolism in cultured human myotubes, while mRNA expression and proteomic analysis showed increased expression of proteins involved in glucose uptake, glycolytic pathways, and the TCA cycle, however, reduced expression of some proteins involved in metabolism of lipids. Similar effects on energy metabolism were observed in osteoblastic cells treated with conditioned medium from myotubes, with increased glucose uptake and reduced oleic acid uptake. Thus, our data may indicate a shift in fuel preference from fatty acid to glucose metabolism in both cell types, induced by conditioned media from the opposite cell type, possibly indicating a more general pattern in communication between these tissues.

## Figures and Tables

**Table 1:** Primers used for qPCR.

Gene	Acc.no.	Forward Sequence	Reverse Sequence
*CD36*	L06850	AGTCACTGCGACATGATTAATGGT	CTGCAATACCTGGCTTTTCTCAA
*GLUT1/SLC2A1*	K03195	CAGCAGCCCTAAGGATCTCTCA	CCGGCTCGGCTGACATC
*MYH2*	C5814	AAGGTCGGCAATGAGTATGTCA	CAACCATCCACAGGAACATCTTC
*MYH7*	NM_000257.2	CTCTGCACAGGGAAAATCTGAA	CCCCTGGACTTTGTCTCATT
*PDK4*	BC040239	TTTCCAGAACCAACCAATTCACA	TGC CCG CAT TGC ATT CTT A
*PGC1* *α*	NM013261.3	AAAGGATGCGCTCTCGTTCA	TCTACTGCCTGGAGACCTTGATC
*GAPDH*	NM002046	TGCACCACCACCTGCTTAGC	GGCATGGACTGTGGTCAT GAG
*RPLP0*	M17885	CCATTCTATCATCAACGGGTACAA	AGCAAGTGGGAAGGTGTAATCC

Acc.no., accession number; *CD36*, cluster of differentiation 36; *GLUT1/SLC2A1*, glucose transporter 1; *MYH2*, myosin heavy chain 2; *MYH7*, myosin heavy chain 7; *PDK4*, pyruvate dehydrogenase kinase 4; PGC1α, peroxisome proliferator-activated receptor gamma coactivator 1-alpha; *GAPDH*, glyceraldehyde 3-phosphate dehydrogenase; *RPLP0*, large ribosomal protein P0. The accession numbers in GenBank were used to design the primers in the Primer Express^®^ program, version 2.0.

**Table 2:** Top 20 most abundant proteins in the conditioned medium from differentiated osteoblastic cells (OB-CM).

	Uniprot ID	Protein Symbol	Protein Name	Biological Process (Gene Ontology)							
1	P06733	ENO1	Alpha-enolase								
2	P62937	PPIA	Peptidyl-prolyl cis-trans isomerase A								
3	P63261	ACTG1	Actin, cytoplasmic 2								
4	P08670	VIM	Vimentin								
5	P09486	SPARC	Secreted protein acidic and cysteine rich								
6	P02765	AHSG	Alpha-2-HS-glycoprotein								
7	P23528	CFL1	Cofilin-1								
8	P09382	LGALS1	Galectin-1								
9	P14618	PKM	Pyruvate kinase								
10	P37802	TAGLN2	Transgelin-2								
11	P68363	TUBA1B	Tubulin alpha-1B chain								
12	O00299	CLIC1	Chloride intracellular channel protein 1								
13	P26022	PTX3	Pentraxin-related protein								
14	P04792	HSPB1	Heat shock protein beta-1								
15	P60709	ACTB	Actin, cytoplasmic 1								
16	P00338	LDHA	L-lactate dehydrogenase A chain								
17	P09211	GSTP1	Glutathione S-transferase P								
18	P07437	TUBB	Tubulin beta chain								
19	P07737	PFN1	Profilin-1								
20	P07355	ANXA2	Annexin 2								

The proteins are classified based on examples of biological process in Gene Ontology. Light green: Cytoskeleton organisation; pink: Regulated exocytosis; yellow: Glycolytic process; blue: Regulation of cellular component organization; grey: Response to cytokine; light blue: Organelle organization; green: Regulation of multi-cellular organismal process; peach coloured: Extracellular matrix organization.

**Table 3:** Top 20 most abundant proteins in the conditioned medium from differentiated myotubes (SKM-CM).

	Uniprot ID	Protein Symbol	Protein Name	Biological Process (Gene Ontology)							
1	P63313	TMSB10	Thymosin beta 10								
2	P62328	TMSB4X	Thymosin beta 4 X-linked								
3	P63261	ACTG1	Actin, cytoplasmic 2								
4	P07737	PFN1	Profilin 1								
5	P02795	MT2A	Metallothionein 2A								
6	P08670	VIM	Vimentin								
7	P09486	SPARC	Secreted protein acidic and cysteine rich								
8	P63104	YWHAZ	Tyrosine 3-monooxygenase/tryptophan 5-monooxygenase activation zeta								
9	P08123	COL1A2	Collagen type I alpha 2 chain								
10	P09382	LGALS1	Galectin 1								
11	P02452	COL1A1	Collagen type I alpha 1 chain								
12	P0DP25	CALM3	Calmodulin 3								
13	P07951	TPM2	Tropomyosin 2								
14	P62258	YWHAE	Tyrosine 3-monooxygenase/tryptophan 5-monooxygenase activation epsilon								
15	Q16270	IGFBP7	Insulin-like growth factor binding protein 7								
16	P17661	DES	Desmin								
17	P41222	PTGDS	Prostaglandin D2 synthase								
18	P10599	TXN	Thioredoxin								
19	P01033	TIMP1	TIMP metallopeptidase inhibitor 1								
20	P06454	PTMA	Prothymosin alpha								

The proteins are classified based on examples of biological process in Gene Ontology. Light green: Cytoskeleton organisation; pink: Regulated exocytosis; yellow: Maintenance of protein location in cell; blue: Regulation of cellular component organization; grey: Response to cytokine; light blue: Organelle organization; green: Regulation of multi-cellular organismal process; peach coloured: Extracellular matrix organization.

**Table 4:** Significantly regulated proteins in common with MitoCarta 3.0.

Uniprot ID	Protein Symbol	Protein Name	Log2 (Fold Change)	*p*-Value
Q16698	DECR1	2,4-dienoyl-CoA reductase 1	0.025562	0.01318
P35914	HMGCL	3-hydroxy-3-methylglutaryl-CoA lyase	0.0276	0.010166
Q99798	ACO2	Aconitase 2	0.010616	0.025911
O75891	ALDH1L1	Aldehyde dehydrogenase 1 family member L1	0.029607	0.004703
P54886	ALDH18A	Aldehyde dehydrogenase 18 family member A1	0.014905	0.048835
P05091	ALDH2	Aldehyde dehydrogenase 2 family member	0.027491	0.006844
Q9Y2R0	COA3	Cytochrome C oxidase assembly factor 3	0.203074	0.032505
P10515	DLAT	Dihydrolipoamide S-acetyltransferase	0.022512	0.046349
P07954	FH	Fumarate hydratase	0.007098	0.047717
P00367	GLUD1	Glutamate dehydrogenase 1	0.014062	0.037031
O94925	GLS	Glutaminase	0.048643	0.021184
Q9Y2Q3	GSTK1	Glutathione S-transferase kappa 1	0.004066	0.031943
Q16836	HADH	Hydroxyacyl-CoA dehydrogenase	0.02538	0.003717
P36776	LONP1	Lon peptidase 1, mitochondrial	0.026353	0.026783
Q8IXM3	MRPL41	Mitochondrial ribosomal protein L41	0.117037	0.021162
Q4G0N4	NADK2	NAD kinase 2, mitochondrial	0.08224	0.0359
P28331	NDUFS1	NADH:Ubiquinone oxidoreductase core subunit S1	0.015448	0.04241
O75251	NDUFS7	NADH:Ubiquinone oxidoreductase core subunit S7	0.033855	0.047395
Q13423	NNT	Nicotinamide nucleotide transhydrogenase	0.012271	0.048518
Q10713	PMPCA	Peptidase, mitochondrial processing subunit alpha	0.029524	0.004
Q9Y3E5	PTRH2	Peptidyl-TRNA hydrolase 2	0.019982	0.029719
P32322	PYCR1	Pyrroline-5-carboxylate reductase 1	0.034958	0.019611
P08559	PDHA1	Pyruvate dehydrogenase E1 subunit alpha 1	0.028673	0.012049
P11177	PDHB	Pyruvate dehydrogenase E1 subunit beta	0.028679	0.008729
P34897	SHMT2	Serine hydroxymethyltransferase 2	0.02749	0.033103
P49411	TUFM	Tu translation elongation factor, mitochondrial	0.010111	0.035421
P07108	DBI	Acyl-CoA binding protein	−0.04511	0.02523
Q15046	KARS1	Lysyl-TRNA synthetase 1	−0.01335	0.035324

## Data Availability

The data presented in this study are available on request from the corresponding author.

## Supplementary Materials

The following supporting information can be downloaded at: https://www.mdpi.com/article/10.3390/biomedicines11112908/s1, Table S1: Secretome OB-CM; Table S2: Secretome SKM-CM; Table S3: Proteome full list.

## References

[1] Kirk B., Feehan J., Lombardi G., Duque G. (2020). Muscle, Bone, and Fat Crosstalk: The Biological Role of Myokines, Osteokines, and Adipokines. Curr. Osteoporos. Rep..

[2] Li G., Zhang L., Wang D., AIQudsy L., Jiang J.X., Xu H., Shang P. (2019). Muscle-bone crosstalk and potential therapies for sarco-osteoporosis. J. Cell. Biochem..

[3] Buvinic S., Balanta-Melo J., Kupczik K., Vasquez W., Beato C., Toro-Ibacache V. (2020). Muscle-Bone Crosstalk in the Masticatory System: From Biomechanical to Molecular Interactions. Front. Endocrinol..

[4] Pedersen B.K. (2011). Muscles and their myokines. J. Exp. Biol..

[5] Severinsen M.C.K., Pedersen B.K. (2020). Muscle-Organ Crosstalk: The Emerging Roles of Myokines. Endocr. Rev..

[6] Pedersen L., Hojman P. (2012). Muscle-to-organ cross talk mediated by myokines. Adipocyte.

[7] Lee J.Y., Park S.J., Han S.A., Lee S.H., Koh J.M., Hamrick M.W., Kim B.J. (2019). The effects of myokines on osteoclasts and osteoblasts. Biochem. Biophys. Res. Commun..

[8] Mera P., Laue K., Wei J., Berger J.M., Karsenty G. (2016). Osteocalcin is necessary and sufficient to maintain muscle mass in older mice. Mol. Metab..

[9] Mera P., Laue K., Ferron M., Confavreux C., Wei J., Galan-Diez M., Lacampagne A., Mitchell S.J., Mattison J.A., Chen Y. (2017). Osteocalcin Signaling in Myofibers Is Necessary and Sufficient for Optimum Adaptation to Exercise. Cell Metab..

[10] Yoshida Y., Jain S.S., McFarlan J.T., Snook L.A., Chabowski A., Bonen A. (2013). Exercise- and training-induced upregulation of skeletal muscle fatty acid oxidation are not solely dependent on mitochondrial machinery and biogenesis. J. Physiol..

[11] Frontera W.R., Ochala J. (2015). Skeletal muscle: A brief review of structure and function. Calcif. Tissue Int..

[12] DeFronzo R.A., Tripathy D. (2009). Skeletal muscle insulin resistance is the primary defect in type 2 diabetes. Diabetes Care.

[13] Civitelli R., Ziambaras K. (2011). Calcium and phosphate homeostasis: Concerted interplay of new regulators. J. Endocrinol. Investig..

[14] DiGirolamo D.J., Kiel D.P., Esser K.A. (2013). Bone and skeletal muscle: Neighbors with close ties. J. Bone Miner. Res..

[15] Lee N.K., Sowa H., Hinoi E., Ferron M., Ahn J.D., Confavreux C., Dacquin R., Mee P.J., McKee M.D., Jung D.Y. (2007). Endocrine regulation of energy metabolism by the skeleton. Cell.

[16] Mengeste A.M., Nikolic N., Dalmao Fernandez A., Feng Y.Z., Nyman T.A., Kersten S., Haugen F., Kase E.T., Aas V., Rustan A.C. (2022). Insight into the Metabolic Adaptations of Electrically Pulse-Stimulated Human Myotubes Using Global Analysis of the Transcriptome and Proteome. Front. Physiol..

[17] Lund J., Rustan A.C., Løvsletten N.G., Mudry J.M., Langleite T.M., Feng Y.Z., Stensrud C., Brubak M.G., Drevon C.A., Birkeland K.I. (2017). Exercise in vivo marks human myotubes in vitro: Training-induced increase in lipid metabolism. PLoS ONE.

[18] Jafari A., Qanie D., Andersen T.L., Zhang Y., Chen L., Postert B., Parsons S., Ditzel N., Khosla S., Johansen H.T. (2017). Legumain Regulates Differentiation Fate of Human Bone Marrow Stromal Cells and Is Altered in Postmenopausal Osteoporosis. Stem Cell Rep..

[19] Wensaas A.J., Rustan A.C., Lovstedt K., Kull B., Wikstrom S., Drevon C.A., Hallen S. (2007). Cell-based multiwell assays for the detection of substrate accumulation and oxidation. J. Lipids Res..

[20] Folch J., Lees M., Sloane Stanley G.H. (1957). A simple method for the isolation and purification of total lipides from animal tissues. J. Biol. Chem..

[21] Batth T.S., Tollenaere M.X., Rüther P., Gonzalez-Franquesa A., Prabhakar B.S., Bekker-Jensen S., Deshmukh A.S., Olsen J.V. (2019). Protein Aggregation Capture on Microparticles Enables Multipurpose Proteomics Sample Preparation. Mol. Cell. Proteom..

[22] Rath S., Sharma R., Gupta R., Ast T., Chan C., Durham T.J., Goodman R.P., Grabarek Z., Haas M.E., Hung W.H.W. (2021). MitoCarta3.0: An updated mitochondrial proteome now with sub-organelle localization and pathway annotations. Nucleic Acids Res..

[23] Perez-Riverol Y., Bai J., Bandla C., Hewapathirana S., García-Seisdedos D., Kamatchinathan S., Kundu D., Prakash A., Frericks-Zipper A., Eisenacher M. (2022). The PRIDE database resources in 2022: A Hub for mass spectrometry-based proteomics evidences. Nucleic Acids Res..

[24] Melouane A., Carbonell A., Yoshioka M., Puymirat J., St-Amand J. (2018). Implication of SPARC in the modulation of the extracellular matrix and mitochondrial function in muscle cells. PLoS ONE.

[25] Melouane A., Yoshioka M., Kanzaki M., St-Amand J. (2019). Sparc, an EPS-induced gene, modulates the extracellular matrix and mitochondrial function via ILK/AMPK pathways in C2C12 cells. Life Sci..

[26] Mathes S., Fahrner A., Luca E., Krutzfeldt J. (2022). Growth hormone/IGF-I-dependent signaling restores decreased expression of the myokine SPARC in aged skeletal muscle. J. Mol. Med..

[27] Hardie D.G. (2011). Energy sensing by the AMP-activated protein kinase and its effects on muscle metabolism. Proc. Nutr. Soc..

[28] Battafarano G., Rossi M., Marampon F., Minisola S., Del Fattore A. (2020). Bone Control of Muscle Function. Int. J. Mol. Sci..

[29] Colaianni G., Storlino G., Sanesi L., Colucci S., Grano M. (2020). Myokines and Osteokines in the Pathogenesis of Muscle and Bone Diseases. Curr. Osteoporos. Rep..

[30] Laurens C., Parmar A., Murphy E., Carper D., Lair B., Maes P., Vion J., Boulet N., Fontaine C., Marquès M. (2020). Growth and differentiation factor 15 is secreted by skeletal muscle during exercise and promotes lipolysis in humans. JCI Insight.

[31] Koshal P., Matera I., Abruzzese V., Ostuni A., Bisaccia F. (2022). The Crosstalk between HepG2 and HMC-III Cells: In Vitro Modulation of Gene Expression with Conditioned Media. Int. J. Mol. Sci..

[32] Vis M.A.M., Ito K., Hofmann S. (2020). Impact of Culture Medium on Cellular Interactions in in vitro Co-culture Systems. Front. Bioeng. Biotechnol..

[33] Alekos N.S., Moorer M.C., Riddle R.C. (2020). Dual Effects of Lipid Metabolism on Osteoblast Function. Front. Endocrinol..

[34] Goodpaster B.H., Sparks L.M. (2017). Metabolic Flexibility in Health and Disease. Cell Metab..

[35] Donat A., Knapstein P.R., Jiang S., Baranowsky A., Ballhause T.M., Frosch K.H., Keller J. (2021). Glucose Metabolism in Osteoblasts in Healthy and Pathophysiological Conditions. Int. J. Mol. Sci..

[36] Shen L., Hu G., Karner C.M. (2022). Bioenergetic Metabolism in Osteoblast Differentiation. Curr. Osteoporos. Rep..

[37] Alghannam A.F., Ghaith M.M., Alhussain M.H. (2021). Regulation of Energy Substrate Metabolism in Endurance Exercise. Int. J. Environ. Res. Public Health.

[38] Son J.S., Chae S.A., Testroet E.D., Du M., Jun H.P. (2018). Exercise-induced myokines: A brief review of controversial issues of this decade. Expert Rev. Endocrinol. Metab..

[39] Norheim F., Raastad T., Thiede B., Rustan A.C., Drevon C.A., Haugen F. (2011). Proteomic identification of secreted proteins from human skeletal muscle cells and expression in response to strength training. Am. J. Physiol. Endocrinol. Metab..

[40] Alquier T., Christian-Hinman C.A., Alfonso J., Faergeman N.J. (2021). From benzodiazepines to fatty acids and beyond: Revisiting the role of ACBP/DBI. Trends Endocrinol. Metab..

[41] Busnelli M., Manzini S., Parolini C., Escalante-Alcalde D., Chiesa G. (2018). Lipid phosphate phosphatase 3 in vascular pathophysiology. Atherosclerosis.

[42] George Warren W., Osborn M., Yates A., Wright K., O’Sullivan S.E. (2023). The emerging role of fatty acid binding protein 5 (FABP5) in cancers. Drug Discov. Today.

[43] Dias J., Octobre G., Kobbi L., Comisso M., Flisiak S., Mirande M. (2012). Activation of human mitochondrial lysyl-tRNA synthetase upon maturation of its premitochondrial precursor. Biochemistry.

[44] Russell A.P. (2005). PGC-1alpha and exercise: Important partners in combating insulin resistance. Curr. Diabetes Rev..

[45] Schiaffino S., Reggiano C. (2011). Fiber types in mammalian skeletal muscles. Physiol. Rev..

